# Association Between Daily Alcohol Intake and Risk of All-Cause Mortality: A Systematic Review and Meta-analyses

**DOI:** 10.1001/jamanetworkopen.2023.6185

**Published:** 2023-03-31

**Authors:** Jinhui Zhao, Tim Stockwell, Tim Naimi, Sam Churchill, James Clay, Adam Sherk

**Affiliations:** 1 Canadian Institute for Substance Use Research, University of Victoria, Victoria, British Columbia, Canada; 2 Department of Psychology, University of Portsmouth, Portsmouth, Hampshire, United Kingdom

## Abstract

**Importance:**

A previous meta-analysis of the association between alcohol use and all-cause mortality found no statistically significant reductions in mortality risk at low levels of consumption compared with lifetime nondrinkers. However, the risk estimates may have been affected by the number and quality of studies then available, especially those for women and younger cohorts.

**Objective:**

To investigate the association between alcohol use and all-cause mortality, and how sources of bias may change results.

**Data Sources:**

A systematic search of PubMed and Web of Science was performed to identify studies published between January 1980 and July 2021.

**Study Selection:**

Cohort studies were identified by systematic review to facilitate comparisons of studies with and without some degree of controls for biases affecting distinctions between abstainers and drinkers. The review identified 107 studies of alcohol use and all-cause mortality published from 1980 to July 2021.

**Data Extraction and Synthesis:**

Mixed linear regression models were used to model relative risks, first pooled for all studies and then stratified by cohort median age (<56 vs ≥56 years) and sex (male vs female). Data were analyzed from September 2021 to August 2022.

**Main Outcomes and Measures:**

Relative risk estimates for the association between mean daily alcohol intake and all-cause mortality.

**Results:**

There were 724 risk estimates of all-cause mortality due to alcohol intake from the 107 cohort studies (4 838 825 participants and 425 564 deaths available) for the analysis. In models adjusting for potential confounding effects of sampling variation, former drinker bias, and other prespecified study-level quality criteria, the meta-analysis of all 107 included studies found no significantly reduced risk of all-cause mortality among occasional (>0 to <1.3 g of ethanol per day; relative risk [RR], 0.96; 95% CI, 0.86-1.06; *P* = .41) or low-volume drinkers (1.3-24.0 g per day; RR, 0.93; *P* = .07) compared with lifetime nondrinkers. In the fully adjusted model, there was a nonsignificantly increased risk of all-cause mortality among drinkers who drank 25 to 44 g per day (RR, 1.05; *P* = .28) and significantly increased risk for drinkers who drank 45 to 64 and 65 or more grams per day (RR, 1.19 and 1.35; *P* < .001). There were significantly larger risks of mortality among female drinkers compared with female lifetime nondrinkers (RR, 1.22; *P* = .03).

**Conclusions and Relevance:**

In this updated systematic review and meta-analysis, daily low or moderate alcohol intake was not significantly associated with all-cause mortality risk, while increased risk was evident at higher consumption levels, starting at lower levels for women than men.

## Key Points

**Question:**

What is the association between mean daily alcohol intake and all-cause mortality?

**Findings:**

This systematic review and meta-analysis of 107 cohort studies involving more than 4.8 million participants found no significant reductions in risk of all-cause mortality for drinkers who drank less than 25 g of ethanol per day (about 2 Canadian standard drinks compared with lifetime nondrinkers) after adjustment for key study characteristics such as median age and sex of study cohorts. There was a significantly increased risk of all-cause mortality among female drinkers who drank 25 or more grams per day and among male drinkers who drank 45 or more grams per day.

**Meaning:**

Low-volume alcohol drinking was not associated with protection against death from all causes.

## Teaser

This systematic review and meta-analysis evaluates the association between daily alcohol intake and risk of all-cause mortality.

## Introduction

The proposition that low-dose alcohol use protects against all-cause mortality in general populations continues to be controversial.^1^ Observational studies tend to show that people classified as “moderate drinkers” have longer life expectancy and are less likely to die from heart disease than those classified as abstainers.^2^ Systematic reviews and meta-analyses of this literature^3^ confirm J-shaped risk curves (protective associations at low doses with increasing risk at higher doses). However, mounting evidence suggests these associations might be due to systematic biases that affect many studies. For example, light and moderate drinkers are systematically healthier than current abstainers on a range of health indicators unlikely to be associated with alcohol use eg, dental hygiene, exercise routines, diet, weight, income^4^; lifetime abstainers may be systematically biased toward poorer health^5^; studies fail to control for biases in the abstainer reference group, in particular failing to remove “sick quitters” or former drinkers, many of whom cut down or stop for health reasons^2^; and most studies have nonrepresentative samples leading to an overrepresentation of older White men. Adjustment of cohort samples to make them more representative has been shown to eliminate apparent protective associations.^6^ Mendelian randomization studies that control for the confounding effects of sociodemographic and environmental factors find no evidence of cardioprotection.^7^

We published 2 previous systematic reviews and meta-analyses that investigated these hypotheses. The first of these focused on all-cause mortality,^8^ finding negligible reductions in mortality risk with low-volume alcohol use when study-level controls were introduced for potential bias and confounding, such as the widespread practice of misclassifying former drinkers and/or current occasional drinkers as abstainers (ie, not restricting reference groups to lifetime abstainers).^8^ Our alcohol and coronary heart disease (CHD) mortality meta-analysis of 45 cohort studies^9^ found that CHD mortality risk differed widely by age ranges and sex of study populations. In particular, young cohorts followed up to old age did not show significant cardio-protection for low-volume use. Cardio-protection was only apparent among older cohorts that are more exposed to lifetime selection biases (ie, increasing numbers of “sick-quitters” in the abstainer reference groups and the disproportionate elimination of drinkers from the study sample who had died or were unwell).

The present study updates our earlier systematic review and meta-analysis for all-cause mortality and alcohol use,^8^ including studies published up to July 2021 (ie, 6.5 years of additional publications). The study also investigated the risk of all-cause mortality for alcohol consumption according to (1) median ages of the study populations (younger than 56 years or 56 years and older), replicating the methods of Zhao et al^9^; (2) the sex distribution of the study populations, and (3) studies of cohorts recruited before a median age of 51 years of age and followed up in health records until a median age of at least 60 years (ie, with stricter rules to further minimize lifetime selection biases). Because younger cohorts followed up to an age at which they may experience heart disease are less likely to be affected by lifetime selection biases,^9^ we hypothesized that such studies would be less likely to show reduced mortality risks for low-volume drinkers. Finally, we reran the analyses using occasional drinkers (<1 drink per week) as the reference, for whom physiological health benefits are unlikely. Occasional drinkers are a more appropriate reference group, given evidence demonstrating that lifetime abstainers may be biased toward ill health.^10^

## Methods

The present study updates the systematic reviews and meta-analyses described above^8^ by including studies published up to July 2021 to investigate whether the risk differed for subgroups. The study protocol was preregistered on the Open Science Framework.^11^ Inclusion criteria, search strategy, study selection, data extraction, and statistical analytical methods of the study are summarized in later sections (see eAppendix in Supplement 1 for more details).

The systematic review followed the Preferred Reporting Items for Systematic Reviews and Meta-analyses (PRISMA) reporting guideline.^12^ The review sought cohort studies of all-cause mortality and alcohol consumption. We identified all potentially relevant articles published up to July 31, 2021, regardless of language, by searching PubMed and Web of Science, through reference list cross-checking of previous meta-analyses (eFigure 1 in Supplement 1). There were 87 studies identified by Stockwell et al.^8^ After inclusion of 20 new studies meeting inclusion criteria, there were a total of 107 cohort studies (eTable 1 in Supplement 1).^13,14,15,16,17,18,19,20,21,22,23,24,25,26,27,28,29,30,31,32^

Three coders (J. Z., F. A., and J. C.) reviewed all eligible studies to extract and code data independently from all studies fulfilling the inclusion criteria. Data extracted included (1) outcome, all-cause mortality; (2) measures of alcohol consumption; (3) study characteristics, including cohort ages at recruitment and follow-up; (4) types of misclassification error of alcohol consumers and abstainers; (5) controlled variables in individual studies. Alcoholic drinks were converted into grams per day according to country-specific definitions if not otherwise defined.^33,34^

### Statistical Analysis

We also assessed publication bias, heterogeneity, and confounding of covariates that might potentially affect the association of interest using several statistical approaches.^35,36,37,38,39,40,41^ Relative risk (RR), including hazard ratios or rate ratios, were converted to natural log-transformed formats to deal with skewness. Publication bias was assessed through visual inspection of the funnel plot of log-RR of all-cause mortality due to alcohol consumption against the inverse standard error of log-RR^42^ and Egger’s linear regression method.^36^ We also plotted forest graphs of log-RR of all-cause mortality for any level of drinking to assess heterogeneity among studies.^42^ The between-study heterogeneity of RRs were assessed using Cochran *Q*^37^ and the *I*^2^ statistic.^38^ If heterogeneity was detected, mixed-effects models were used to obtain the summarized RR estimates. Mixed-effects regression analyses were performed in which drinking groups and control variables were treated as fixed-effects with a random study effect because of significant heterogeneity.^43^

All analyses were weighted by the inverse of the estimated variance of the natural log relative risk. Variance was estimated from reported standard errors, confidence intervals, or number of deaths. The weights for each individual study were created using the inverse variance weight scheme and used in mixed regression analysis to get maximum precision for the main results of the meta-analysis.^42^ In comparison with lifetime abstainers, the study estimated the mean RR of all-cause mortality for former drinkers (ie, now completely abstaining), current occasional (<9.1 g per week), low-volume (1.3-24.0 g per day), medium-volume (25.0-44.0 g per day), high-volume (45.0-64.0 g) and highest-volume drinkers (≥65.0 grams per day). The analyses adjusted for the potential confounding effects of study characteristics including the median age and sex distribution of study samples, drinker biases, country where a study was conducted, follow-up years and presence or absence of confounders. Analyses were also repeated using occasional drinkers as the reference group. We used *t* tests to calculate *P* values, and significance was set at .05. All statistical analyses were performed using SAS version 9.4 (SAS Institute) and the SAS MIXED procedure was used to model the log-transformed RR.^44^ Data were analyzed from September 2021 to August 2022.

## Results

### Characteristics of Included Studies

There were 724 estimates of the risk relationship between level of alcohol consumption and all-cause mortality from 107 unique studies^13,14,15,16,17,18,19,20,21,22,23,24,25,26,27,28,29,30,31,32,45,46,47,48,49,50,51,52,53,54,55,56,57,58,59,60,61,62,63,64,65,66,67,68,69,70,71,72,73,74,75,76,77,78,79,80,81,82,83,84,85,86,87,88,89,90,91,92,93,94,95,96,97,98,99,100,101,102,103,104,105,106,107,108,109,110,111,112,113,114,115,116,117,118,119,120,121,122,123,124,125,126,127,128,129,130,131^, including 4 838 825 participants and 425 564 deaths available for the analysis. Table 1 describes the sample characteristics of the metadata. Of 39 studies^13,15,18,21,23,24,25,26,29,31,45,46,47,49,50,52,53,54,57,58,59,62,64,70,80,81,85,87,91,94,96,100,104,107,118,124,125,127,130^reporting RR estimates for men and women separately, 33^14,17,48,51,61,63,66,68,69,72,76,79,83,84,86,88,90,92,93,97,98,101,103,105,109,110,111,113,114,115,119,120,128^ were for males only, 8^16,65,73,99,102,108,112,123^ for females only, and 30^13,19,20,21,22,26,27,28,29,30,32,55,56,67,71,74,75,77,78,82,84,89,95,106,116,117,121,122,126,129^ for both sexes. Twenty-one studies^13,17,19,21,22,26,27,45,46,47,48,49,50,51,52,53,54,55,56,57,58^ (220 risk estimates) were free from abstainer bias (ie, had a reference group of strictly defined lifetime abstainers). There were 50 studies^14,15,16,18,20,23,24,25,29,59,60,61,62,63,64,65,66,67,68,69,70,71,72,73,74,75,76,77,78,79,80,81,82,83,84,85,86,87,88,89,90,91,92,93,94,95,96,97,98,99^ (265 risk estimates) with both former and occasional drinker bias; 28 studies^28,30,31,32,100,101,102,103,104,105,106,107,108,109,110,111,112,113,114,115,116,117,118,119,120,121,122,130^ (177 risk estimates) with only former drinker bias; and 8 studies^123,124,125,126,127,128,129,131^ (62 risk estimates) with only occasional drinker bias.

**Table 1.** The Sample Characteristics of the Metadata on All-Cause Mortality and Alcohol Consumption From 1980 to 2022

Covariates	All-cause mortality studies (n = 107)		Mean RR (95% CI)^b^	*P* value
	Studies, No. (%)	No. of RRs (%)^a^		
Publication year				
1980-2004	51 (47.66)	310 (42.82)	1.08 (1.03-1.14)	.26
2005-2014	36 (33.64)	220 (30.39)	1.07 (1.00-1.13)	.44
2015-2021^c^	20 (18.69)	194 (26.80)	1.03 (0.96-1.10)	NA
Median age, y^d^				
19-55	67 (59.29)	438 (60.50)	1.10 (1.05-1.14)	.02
56-78^c^	46 (40.71)	286 (39.50)	1.01 (0.96-1.07)	NA
Sex				
Men only	73 (48.03)	343 (47.38)	1.05 (1.00-1.11)	.47
Women only	48 (31.58)	226 (31.22)	1.11 (1.05-1.17)	.08
Men and women^c^	31 (20.39)	155 (21.41)	1.02 (0.94-1.10)	NA
Countries				
North and Central America	41 (37.96)	287 (39.64)	1.05 (1.00-1.11)	.58
Europe and Australia	53 (49.07)	343 (47.38)	1.07 (1.02-1.12)	.79
Asia^c^	14 (12.96)	94 (12.98)	1.08 (0.98-1.18)	NA
Follow-up years				
03.70-10.50^c^	45 (42.06)	266 (36.74)	0.98 (0.92-1.03)	NA
11.00-41.00	62 (57.94)	458 (63.26)	1.12 (1.07-1.16)	<.001
Baseline conditions^e^				
Exclusion/control	49 (45.37)	342 (47.24)	1.12 (1.07-1.17)	.004
No exclusion/control	59 (54.63)	382 (52.76)	1.01 (0.97-1.06)	
Alcohol use measure				
Quantity-frequency	30 (28.04)	222 (30.66)	1.18 (1.12-1.25)	<.001
Others^c^	77 (71.96)	502 (69.34)	1.01 (0.97-1.05)	NA
Abstainer biases				
Both former and occasional	50 (46.73)	265 (36.60)	0.98 (0.92-1.04)	<.001
Former drinker bias only	28 (26.17)	177 (24.45)	1.05 (0.98-1.12)	.005
Occasional bias only	8 (7.48)	62 (8.56)	1.01 (0.89-1.13)	.009
Neither bias^c^^,^^f^	21 (19.63)	220 (30.39)	1.19 (1.12-1.25)	NA
Control for smoking^g^				
No	18 (16.51)	135 (18.65)	1.09 (1.01-1.18)	.43
Yes^c^	91 (83.49)	589 (81.35)	1.06 (1.02-1.10)	NA
Control for SES				
No	39 (36.11)	245 (33.84)	1.16 (1.10-1.22)	<.001
Yes^c^	69 (63.89)	479 (66.16)	1.01 (0.97-1.06)	NA
Control for race				
No	79 (73.83)	530 (73.20)	1.07 (1.02-1.11)	.85
Yes^c^	28 (26.17)	194 (26.80)	1.06 (0.99-1.13)	NA
Control for diet				
No	90 (84.11)	570 (78.73)	1.06 (1.02-1.10)	.84
Yes^c^	17 (15.89)	154 (21.27)	1.07 (0.99-1.15)	NA
Control for exercise				
No	63 (58.88)	431 (59.53)	1.04 (1.00-1.09)	.13
Yes^c^	44 (41.12)	293 (40.47)	1.10 (1.04-1.15)	NA
Control for BMI^h^				
No	48 (44.04)	294 (40.61)	1.09 (1.03-1.14)	.28
Yes^c^	61 (55.96)	430 (59.39)	1.05 (1.00-1.09)	NA

Abbreviations: BMI, body mass index; NA, not applicable; RR, relative risk; SES, socioeconomic status.

^a^ Number of relative risk estimates for any drinking from the included studies.

^b^ Unadjusted mean relative risk (RR) and 95% confidence interval (CI) due to any drinking from the included studies.

^c^ Reference category.

^d^ Median age at study enrollment.

^e^ Control for heart disease and/or other illnesses.

^f^ Lifetime abstention was strictly defined as 0 consumption or never drank 1 drink and did not include studies with any level of occasional lifetime or past year drinking (eg, less than 12 drinks or “rarely” or “hardly ever” drinking).

^g^ Smoking confounding effect was adjusted for in multivariable regression analysis in original studies.

^h^ Body mass index is calculated as weight in kilograms divided by height in meters squared.

Unadjusted mean RR estimates for most study subgroups categorized by methods/sample characteristics showed markedly or significantly higher RRs for alcohol consumers as a group vs abstainers. Exceptions were for studies with less than 10 years of follow-up and those with some form of abstainer bias (Table 1). Bivariable analyses showed that mortality risks for alcohol consumers varied considerably according to other study characteristics, such as quality of the alcohol consumption measure, whether unhealthy individuals were excluded at baseline, and whether socioeconomic status was controlled for (Table 1).

No evidence of publication bias was detected either by inspection of symmetry in the funnel plot of log-RR estimates and their inverse standard errors (eFigure 2 in Supplement 1) or by Egger linear regression analysis (eTable 2 in Supplement 1, all P > .05 for each study group). Significant heterogeneity was observed across studies for all drinking categories confirmed by both the *Q* statistic (*Q*_723_ = 5314.80; *P* < .001) and *I*^2^ estimates (all >85.87%). (See eFigure 3 in Supplement 1 for forest plot of unadjusted risk estimates of mortality risks for the 20 newly identified studies).

### All-Cause Mortality Risk for Drinkers From Meta-analysis of Pooled Studies

Pooled unadjusted estimates (724 observations) showed significantly higher risk for former drinkers (RR, 1.22; 95% CI, 1.11-1.33; *P* = .001) and significantly lower risk for low-volume drinkers (RR, 0.85; 95% CI, 0.81-0.88; *P* = .001) compared with abstainers as defined in the included studies (Table 2; eFigure 4 in Supplement 1). In the fully adjusted model, mortality RR estimates increased for all drinking categories, becoming nonsignificant for low-volume drinkers (RR, 0.93; 95% CI, 0.85-1.01; *P* = .07), occasional drinkers (>0 to <1.3 g of ethanol per day; RR, 0.96; 95% CI, 0.86-1.06; *P* = .41), and drinkers who drank 25 to 44 g per day (RR, 1.05; 95% CI, 0.96-1.14; *P* = .28). There was a significantly increased risk among drinkers who drank 45 to 64 g per day (RR, 1.19; 95% CI, 1.07-1.32; *P* < .001) and 65 or more grams (RR, 1.35; 95% CI, 1.23-1.47; *P* < .001). The Figure shows the changes in RR estimates for low-volume drinkers when removing each covariate from the fully adjusted model. In most cases, removing study-level covariates tended to yield lower risk estimates from alcohol use.

**Table 2.** Mean Relative Risk Estimates of All-Cause Mortality Due to Alcohol Consumption Up to 2022 According to 107 Studies With 724 Relative Risk Estimates

Drinking categories	Studies, No./risk estimates, No.	Unadjusted^a^		Partially adjusted^b^			Fully adjusted^c^	
		RR (95% CI)	*P* value	RR (95% CI)		*P* value	RR (95% CI)	*P* value
Reference group = lifetime nondrinker								
Abstainer	107/191	1 [Reference]		1 [Reference]			1 [Reference]	
Any drinker vs abstainer	107/724	1.06 (0.90-1.25)	.42	1.03 (0.89-1.19)		.65	1.11 (0.96-1.28)	.12
Former drinker vs abstainer	28/56	1.22 (1.11-1.33)	<.001	1.17 (1.08-1.26)		<.001	1.26 (1.12-1.42)	.0001
Active drinker vs abstainer, g/d	107/668	0.97 (0.94-1.00)	.02	0.93 (0.90-0.96)		<.001	1.02 (0.93-1.13)	.61
Occasional (<1.30)	24/57	0.92 (0.84-1.01)	.08	0.89 (0.83-0.95)		<.001	0.96 (0.86-1.06)	.41
Low-volume (1.30 to <25)	99/306	0.85 (0.81-0.88)	<.001	0.86 (0.83-0.88)		<.001	0.93 (0.85-1.01)	.08
Medium volume (25 to <45)	80/146	1.02 (0.96-1.08)	.55	0.97 (0.92-1.02)		.21	1.05 (0.96-1.14)	.28
High volume (45 to <65)	52/76	1.07 (0.99-1.16)	.09	1.11 (1.03-1.21)		.009	1.19 (1.07-1.32)	.001
Higher volume (≥65)	45/83	1.35 (1.26-1.46)	<.001	1.24 (1.16-1.32)		<.001	1.35 (1.23-1.47)	.0001
Reference group = occasional drinker								
Abstainer		1.09 (0.99-1.19)	.07	1.12 (1.05-1.20)		<.001	1.04 (0.94-1.16)	.45
Any drinker vs occasional drinker	107/724	1.15 (0.95-1.39)	.14	1.16 (0.99-1.36)		.08	1.16 (0.97-1.38)	.11
Former drinker vs abstainer	28/56	1.33 (1.18-1.50)	<.001	1.31 (1.19-1.46)		<.001	1.31 (1.13-1.52)	.0007
Active drinker vs abstainer, g/d	107/668	1.05 (0.96-1.16)	.29	1.04 (0.97-1.13)		.25	1.06 (0.92-1.23)	.41
Occasional (<1.30)	24/57	1 [Reference]	NA	1 [Reference]		NA	1 [Reference]	
Low-volume (1.30 to <25)	99/306	0.92 (0.84-1.02)	.12	0.97 (0.90-1.04)		.36	0.97 (0.85-1.11)	.65
Medium volume (25 to <45)	80/146	1.11 (0.99-1.24)	.07	1.09 (1.00-1.19)		.047	1.09 (0.96-1.25)	.19
High volume (45 to <65)	52/76	1.16 (1.03-1.31)	.02	1.25 (1.12-1.39)		<.001	1.24 (1.07-1.44)	.004
Higher volume (≥65)	45/83	1.47 (1.30-1.65)	<.001	1.39 (1.27-1.53)		<.001	1.41 (1.23-1.61)	.0001

Abbreviations: NA, not applicable; RR, relative risk.

^a^ Natural log of the RR estimated using the rate ratio or hazard ratio without weighting and adjusting for between-study variation or covariates.

^b^ Weighted estimates adjusted for between-study variation.

^c^ Weighted estimates adjusted for between-study variation, abstainer biases, median age, sex, country in which a study was conducted, study publication year, follow-up years of study samples, drinking pattern, and whether studies controlled for heart problem, social status, race, diet, exercise, body mass index, and smoking status.

**Figure.**
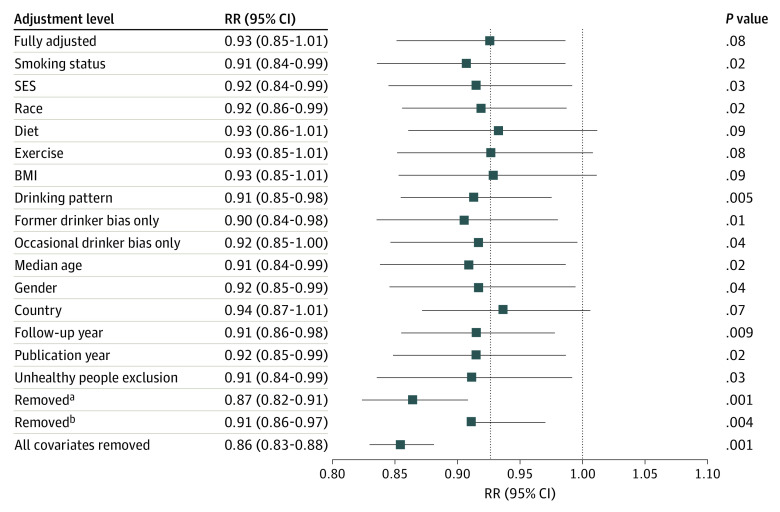
Relative Risk (RR) of All-Cause Mortality Due to Low-Volume Alcohol Consumption (1.3-24.0 g Ethanol per Day) With and Without Adjustment for Potential Confounding by Each Covariate or Set of Covariates BMI indicates body mass index; SES, socioeconomic status. ^a^Variables smoking status, SES, drinking pattern, former drinker bias only, occasional drinker bias, median age, and gender were removed. ^b^Variables race, diet, exercise, BMI, country, follow-up year, publication year, and unhealthy people exclusion were removed.

Table 2 presents the RR estimates when occasional drinkers were the reference group. In fully adjusted models, higher though nonsignificant mortality risks were observed for both abstainers and medium-volume drinkers (RR, 1.04; 95% CI, 0.94-1.16; *P* = .44 and RR, 1.09; 95% CI, 0.96-1.25; *P* = .19, respectively). There were significantly elevated risks for both high and higher volume drinkers (RR, 1.24; 95% CI, 1.07-1.44; *P* = .004 and RR, 1.41; 95% CI, 1.23-1.61; .*P* = 001, respectively).

### All-Cause Mortality Risk on the Basis of Median Age of Study Cohorts at Baseline

As hypothesized, there was a significant interaction between cohort age and mortality risk (*P* = .02; *F*_601_ = 2.93) and so RR estimates for drinkers were estimated in analyses stratified by median age of the study populations at enrollment (Table 3). In unadjusted and partially adjusted analyses, older cohorts displayed larger reductions in mortality risk associated with low-volume consumption than younger cohorts. However, in fully adjusted analyses with multiple covariates included for study characteristics, these differences disappeared. Younger cohorts also displayed greater mortality risks than older cohorts at higher consumption levels. Among studies in which participants were recruited at age 50 years or younger and followed up to age 60 years (ie, there was likely reduced risk of lifetime selection bias) higher RR estimates were observed for all drinking groups vs lifetime abstainers. These differences were significant in all drinking groups except low-volume drinkers (eTable 3 in Supplement 1).

**Table 3.** Mean RR Estimates of All-Cause Mortality Due to Alcohol Consumption by Median Age at Enrollment of Studies (<56 or ≥56 y) Up to 2022

Drinking categories by median age at enrollment in included studies	Studies, No./risk estimates, No.	Unadjusted^a^		Partially adjusted^b^		Fully adjusted^c^	
		RR (95% CI)	*P* value	RR (95% CI)	*P* value	RR (95% CI)	*P* value
Median age, younger than 56 y							
Abstainer	NA	1 [Reference]	NA	1 [Reference]	NA	1 [Reference]	NA
Any drinker vs abstainer	67/436	1.08 (0.91-1.28)	.32	1.06 (0.93-1.22)	.30	1.13 (0.98-1.30)	.07
Former drinker vs abstainer	12/27	1.26 (1.11-1.42)	<.001	1.16 (1.06-1.26)	.001	1.23 (1.08-1.39)	.001
Active drinker vs abstainer, g/d	67/411	1.00 (0.96-1.03)	.93	0.96 (0.92-1.00)	.04	1.05 (0.97-1.13)	.26
Occasional (<1.30)	14/31	0.89 (0.80-1.01)	.06	0.96 (0.90-1.03)	.22	1.02 (0.94-1.11)	.62
Low-volume (1.30 to <25)	61/202	0.88 (0.84-0.93)	<.001	0.88 (0.84-0.91)	<.001	0.93 (0.86-1.01)	.10
Medium volume (25 to <45)	49/91	1.10 (1.03-1.18)	.005	1.00 (0.93-1.06)	.91	1.06 (0.96-1.16)	.25
High volume (45 to <65)	25/34	1.03 (0.92-1.15)	.60	1.16 (1.05-1.29)	.004	1.24 (1.10-1.40)	<.001
Higher volume (≥65)	28/53	1.39 (1.27-1.53)	<.001	1.29 (1.19-1.41)	<.001	1.38 (1.23-1.55)	<.001
Median age ≥56 y							
Abstainer	NA	1 [Reference]	NA	1 [Reference]	NA	1 [Reference]	NA
Any drinker vs abstainer	46/281	1.02 (0.85-1.22)	.79	0.99 (0.84-1.18)	.94	1.09 (0.94-1.27)	.19
Former drinker vs abstainer	16/29	1.18 (1.04-1.34)	.01	1.17 (1.06-1.29)	.002	1.27 (1.09-1.48)	.002
Active drinker vs abstainer, g/d	46/257	0.92 (0.88-0.97)	.001	0.91 (0.87-0.95)	<.001	1.02 (0.91-1.15)	.75
Occasional (<1.30)	12/26	0.95 (0.83-1.10)	.51	0.83 (0.77-0.90)	<.001	0.91 (0.82-1.01)	.09
Low-volume (1.30 to <25)	44/104	0.79 (0.74-0.84)	<.001	0.84 (0.81-0.87)	<.001	0.93 (0.85-1.02)	.11
Medium volume (25 to <45)	36/55	0.89 (0.81-0.98)	.02	0.95 (0.88-1.02)	.14	1.04 (0.95-1.14)	.37
High volume (45 to <65)	28/42	1.10 (0.99-1.23)	.08	1.05 (0.95-1.16)	.39	1.15 (1.02-1.30)	.02
Higher volume (≥65)	18/30	1.29 (1.13-1.47)	<.001	1.20 (1.10-1.31)	<.001	1.32 (1.18-1.47)	<.001

Abbreviations: NA, not applicable; RR, relative risk.

^a^ Natural log of the RR estimated using the rate ratio or hazard ratio without weighting and adjusting for between-study variation or covariates.

^b^ Weighted estimates adjusted for between-study variation.

^c^ Weighted estimates adjusted for between-study variation, abstainer biases, sex, country in which a study was conducted, study publication year, follow-up years, drinking pattern, and whether studies controlled for heart problem, social status, race, diet, exercise, body mass index, and smoking status.

### All-Cause Mortality Risk for Drinkers by Sex

Across all levels of alcohol consumption, female drinkers had a higher RR of all-cause mortality than males (*P* for interaction  = .001). As can be seen in Table 4, all female drinkers had a significantly increased mortality risk compared with female lifetime nondrinkers (RR, 1.22; 95% CI, 1.02-1.46; *P* = .03). Compared with lifetime abstainers, there was significantly increased risk of all-cause mortality among male drinkers who drank 45 to 64 g per day (RR, 1.15; 95% CI, 1.03-1.28; *P* = .01) and drank 65 or more (RR, 1.34; 95% CI, 1.23-1.47; *P* < .001), and among female drinkers who drank 25 to 44 g per day (RR, 1.21; 95% CI, 1.08-1.36; *P* < .01), 45 to 64 g (RR, 1.34; 95% CI, 1.11-1.63; *P* < .01) and 65 or more grams (RR, 1.61; 95% CI, 1.44-1.80; *P* = .001).

**Table 4.** Mean RRs of All-Cause Mortality Due to Alcohol Consumption by Sex (Men or Women) Up to 2022

Drinking categories by median age	Studies, No./risk estimates, No.	Unadjusted^a^			Partially adjusted^b^		Fully adjusted^c^	
		RR (95% CI)	*P* value		RR (95% CI)	*P* value	RR (95% CI)	*P* value
**Men**								
Abstainer	NA	1 [Reference]	NA		1 [Reference]	NA	1 [Reference]	NA
Any drinker vs abstainer	73/343	1.05 (0.88-1.24	.52		1.05 (0.89-1.22)	.49	1.12 (0.95-1.34)	.14
Former drinker vs abstainer	20/24	1.24 (1.08-1.42)	<.001		1.29 (1.20-1.39)	<.001	1.39 (1.21-1.58)	<.001
Active drinker vs abstainer, g/d	73/319	0.97 (0.93-1.01)	.09		0.96 (0.92-1.00)	.05	1.05 (0.96-1.15)	.27
Occasional (<1.30)	13/15	0.95 (0.80-1.13)	.58		0.93 (0.85-1.01)	.07	1.00 (0.91-1.09)	.97
Low-volume (1.30 to <25)	66/141	0.84 (0.80-0.89)	<.001		0.87 (0.84-0.91)	<.001	0.94 (0.88-1.01)	.07
Medium volume (25 to <45)	54/70	0.97 (0.89-1.05)	.43		0.94 (0.90-0.98)	.008	1.01 (0.93-1.10)	.81
High volume (45 to <65)	37/41	1.01 (0.91-1.12)	.87		1.07 (1.01-1.12)	.01	1.15 (1.03-1.28)	.01
Higher volume (≥65)	36/52	1.35 (1.23-1.48)	<.001		1.25 (1.16-1.32)	<.001	1.34 (1.23-1.47)	<.001
**Women**								
Abstainer	NA	1 [Reference]	NA		1 [Reference]	NA	1 [Reference]	NA
Any drinker vs abstainer	48/226	1.12 (0.88-1.44)	.28		1.03 (0.85-1.26)	.69	1.22 (1.02-1.46)	.04
Former drinker vs abstainer	16/22	1.16 (0.98-1.37)	.08		1.09 (1.03-1.14)	.001	1.27 (1.13-1.43)	<.001
Active drinker vs abstainer, g/d	47/204	0.99 (0.93-1.05)	.64		0.88 (0.84-0.92)	<.001	1.03 (0.92-1.15)	.65
Occasional (<1.30)	15/25	0.87 (0.74-1.01)	.08		0.83 (0.78-0.88)	<.001	0.99 (0.87-1.11)	.82
Low-volume (1.30 to <25)	45/106	0.87 (0.81-0.94)	<.001		0.84 (0.80-0.89)	<.001	0.99 (0.90-1.10)	.90
Medium volume (25 to <45)	37/42	1.16 (1.03-1.31)	.01		1.03 (0.96-1.11)	.44	1.21 (1.08-1.36)	.001
High volume (45 to <65)	17/19	1.12 (0.94-1.34)	.21		1.13 (0.95-1.35)	.15	1.34 (1.11-1.63)	.003
Higher volume (≥65)	11/12	1.77 (1.41-2.21)	<.001		1.37 (1.28-1.47)	<.001	1.61 (1.44-1.80)	<.001

Abbreviations: NA, not applicable; RR, relative risk.

^a^ Natural log of the RR estimated using the rate ratio or hazard ratio without weighting and adjusting for between-study variation or covariates.

^b^ Weighted estimates adjusted for between-study variation.

^c^ Weighted estimates adjusted for between-study variation, abstainer biases, median age, country in which a study was conducted, study publication year, follow-up years, drinking pattern, and whether studies controlled for heart problem, social status, race, diet, exercise, body mass index, and smoking status.

## Discussion

In fully adjusted, prespecified models that accounted for effects of sampling, between-study variation, and potential confounding from former drinker bias and other study-level covariates, our meta-analysis of 107 studies found (1) no significant protective associations of occasional or low-volume drinking (moderate drinking) with all-cause mortality; and (2) an increased risk of all-cause mortality for drinkers who drank 25 g or more and a significantly increased risk when drinking 45 g or more per day.

Several meta-analytic strategies were used to explore the role of abstainer reference group biases caused by drinker misclassification errors and also the potential confounding effects of other study-level quality covariates in studies.^2^ Drinker misclassification errors were common. Of 107 studies identified, 86 included former drinkers and/or occasional drinkers in the abstainer reference group, and only 21 were free of both these abstainer biases. The importance of controlling for former drinker bias/misclassification is highlighted once more in our results which are consistent with prior studies showing that former drinkers have significantly elevated mortality risks compared with lifetime abstainers.

In addition to presenting our fully adjusted models, a strength of the study was the examination of the differences in relative risks according to unadjusted and partially adjusted models, including the effect of removing individual covariates from the fully adjusted model. We found evidence that abstainer biases and other study characteristics changed the shape of the risk relationship between mortality and rising alcohol consumption, and that most study-level controls increased the observed risks from alcohol, or attenuated protective associations at low levels of consumption such that they were no longer significant. The reduced RR estimates for occasional or moderate drinkers observed without adjustment may be due to the misclassification of former and occasional drinkers into the reference group, a possibility which is more likely to have occurred in studies of older cohorts which use current abstainers as the reference group. This study also demonstrates the degree to which observed associations between consumption and mortality are highly dependent on the modeling strategy used and the degree to which efforts are made to minimize confounding and other threats to validity.

It also examined risk estimates when using occasional drinkers rather than lifetime abstainers as the reference group. The occasional drinker reference group avoids the issue of former drinker misclassification that can affect the abstainer reference group, and may reduce confounding to the extent that occasional drinkers are more like low-volume drinkers than are lifetime abstainers.^2,8,132^ In the unadjusted and partially adjusted analyses, using occasional drinkers as the reference group resulted in nonsignificant protective associations and lower point estimates for low-volume drinkers compared with significant protective associations and higher point estimates when using lifetime nondrinkers as the reference group. In the fully adjusted models, there were nonsignificant protective associations for low-volume drinkers whether using lifetime abstainers or occasional drinkers as the reference group, though this was only a RR of 0.97 for the latter.

Across all studies, there were few differences in risk for studies when stratified by median age of enrollment above or below age 56 years in the fully adjusted analyses. However, in the subset of studies who enrolled participants aged 50 years or younger who were followed for at least 10 years, occasional drinkers and medium-volume drinkers had significantly increased risk of mortality and substantially higher risk estimates for high- and higher-volume consumption compared with results from all studies. This is consistent with our previous meta-analysis for CHD,^9^ in which younger cohorts followed up to older age did not show a significantly beneficial association of low-volume consumption, while older cohorts, with more opportunity for lifetime selection bias, showed marked, significant protective associations.

Our study also found sex differences in the risk of all-cause mortality. A larger risk of all-cause mortality for women than men was observed when drinking 25 or more grams per day, including a significant increase in risk for medium-level consumption for women that was not observed for men. However, mortality risk for mean consumption up to 25 g per day were very similar for both sexes.

### Limitations

A number of limitations need to be acknowledged. A major limitation involves imperfect measurement of alcohol consumption in most included studies, and the fact that consumption in many studies was assessed at only 1 point in time. Self-reported alcohol consumption is underreported in most epidemiological studies^133,134^ and even the classification of drinkers as lifetime abstainers can be unreliable, with several studies in developed countries finding that the majority of self-reported lifetime abstainers are in fact former drinkers.^135,136^ If this is the case, the risks of various levels of alcohol consumption relative to presumed lifetime abstainers are underestimates. Merely removing former drinkers from analyses may bias studies in favor of drinkers, since former drinkers may be unhealthy, and should rightly be reallocated to drinking groups according to their history. However, this has only been explored in very few studies. Our study found that mortality risk differed significantly by cohort age and sex. It might be that the risk is also higher for other subgroups, such as people living with HIV,^137^ a possibility future research should investigate.

The number of available studies in some stratified analyses was small, so there may be limited power to control for potential study level confounders. However, the required number of estimates per variable for linear regression can be much smaller than in logistic regression, and a minimum of at least 2 estimates per variable is recommended for linear regression analysis,^138^ suggesting the sample sizes were adequate in all models presented. It has been demonstrated that a pattern of binge (ie, heavy episodic) drinking removes the appearance of reduced health risks even when mean daily volume is low.^139^ Too few studies adequately controlled for this variable to investigate its association with different outcomes across studies. Additionally, our findings only apply to the net effect of alcohol at different doses on all-cause mortality, and different risk associations likely apply for specific disease categories. The biases identified here likely apply to estimates of risk for alcohol and all diseases. It is likely that correcting for these biases will raise risk estimates for many types of outcome compared with most existing estimates.

## Conclusions

This updated meta-analysis did not find significantly reduced risk of all-cause mortality associated with low-volume alcohol consumption after adjusting for potential confounding effects of influential study characteristics. Future longitudinal studies in this field should attempt to minimize lifetime selection biases by not including former and occasional drinkers in the reference group, and by using younger cohorts (ie, age distributions that are more representative of drinkers in the general population) at baseline.

## Notes

Supplement 1. **eAppendix.** Methodology of Meta-analysis on All-Cause Mortality and Alcohol Consumption
eReferences
**eFigure 1.** Flowchart of Systematic Search Process for Studies of Alcohol Consumption and Risk of All-Cause Mortality **eTable 1.** Newly Included 20 Studies (194 Risk Estimates) of All-Cause Mortality and Consumption in 2015 to 2022 **eFigure 2.** Funnel Plot of Log-Relative Risk (In(RR)) of All-Cause Mortality Due to Alcohol Consumption Against Inverse of Standard Error of In(RR) **eFigure 3.** Relative Risk (95% CI) of All-Cause Mortality Due to Any Alcohol Consumption Without Any Adjustment for Characteristics of New Studies Published between 2015 and 2022 **eFigure 4.** Unadjusted, Partially Adjusted, and Fully Adjusted Relative Risk (RR) of All-Cause Mortality for Drinkers (vs Nondrinkers), 1980 to 2022 **eTable 2.** Statistical Analysis of Unadjusted Mean Relative Risk (RR) of All-Cause Mortality for Different Categories of Drinkers for Testing Publication Bias and Heterogeneity of RR Estimates From Included Studies **eTable 3.** Mean Relative Risk (RR) Estimates of All-Cause Mortality Due to Alcohol Consumption up to 2022 for Subgroups (Cohorts Recruited 50 Years of Age or Younger and Followed up to 60 Years of Age)

Supplement 2.
Data Sharing Statement
