# Errors in Figure and Supplement

**DOI:** 10.1001/jamanetworkopen.2023.15283

**Published:** 2023-05-09

In the Original Investigation titled “Association Between Daily Alcohol Intake and Risk of All-Cause Mortality: A Systematic Review and Meta-analyses,”^1^ published March 31, 2023, incorrect 95% CIs were shown in the Figure and Supplement. This article has been corrected.^1^
