# Supplementary material for: Association Between Daily Alcohol Intake and Risk of All-Cause Mortality: A Systematic Review and Meta-analyses
Source: JAMA Netw Open. 2023 Mar 31;6(3):e236185. doi: 10.1001/jamanetworkopen.2023.6185 (PMC10066463; doi:10.1001/jamanetworkopen.2023.6185)
Supplement: Supplement 1. — eAppendix. Methodology of Meta-analysis on All-Cause Mortality and Alcohol Consumption eReferences eFigure 1. Flowchart of Systematic Search Process for Studies of Alcohol Consumption and Risk of All-Cause Mortality eTable 1. Newly Included 20 Studies (194 Risk Estimates) of All-Cause Mortality and Consumption in 2015 to 2022 eFigure 2. Funnel Plot of Log-Relative Risk (In(RR)) of All-Cause Mortality Due to Alcohol Consumption Against Inverse of Standard Error of In(RR) eFigure 3. Relative Risk (95% CI) of All-Cause Mortality Due to Any Alcohol Consumption Without Any Adjustment for Characteristics of New Studies Published between 2015 and 2022 eFigure 4. Unadjusted, Partially Adjusted, and Fully Adjusted Relative Risk (RR) of All-Cause Mortality for Drinkers (vs Nondrinkers), 1980 to 2022 eTable 2. Statistical Analysis of Unadjusted Mean Relative Risk (RR) of All-Cause Mortality for Different Categories of Drinkers for Testing Publication Bias and Heterogeneity of RR Estimates From Included Studies eTable 3. Mean Relative Risk (RR) Estimates of All-Cause Mortality Due to Alcohol Consumption up to 2022 for Subgroups (Cohorts Recruited 50 Years of Age or Younger and Followed up to 60 Years of Age) [file jamanetwopen-e236185-s001.pdf]

Supplemental Online Content

Zhao J, Stockwell T, Naimi T, Churchill S, Clay J, Sherk A. Association between daily alcohol intake and risk of all-cause mortality. *JAMA Netw Open*. 2023;6(3):e236185. doi:10.1001/jamanetworkopen.2023.6185

eAppendix. Methodology of Meta-analysis on All-Cause Mortality and Alcohol Consumption

eReferences

eFigure 1. Flowchart of Systematic Search Process for Studies of Alcohol Consumption and Risk of All-Cause Mortality

eTable 1. Newly Included 20 Studies (194 Risk Estimates) of All-Cause Mortality and Consumption in 2015 to 2022

eFigure 2. Funnel Plot of Log-Relative Risk (In(RR)) of All-Cause Mortality Due to Alcohol Consumption Against Inverse of Standard Error of In(RR)

eFigure 3. Relative Risk (95% CI) of All-Cause Mortality Due to Any Alcohol Consumption Without Any Adjustment for Characteristics of New Studies Published between 2015 and 2022

eFigure 4. Unadjusted, Partially Adjusted, and Fully Adjusted Relative Risk (RR) of All-Cause Mortality for Drinkers (vs Nondrinkers), 1980 to 2022

eTable 2. Statistical Analysis of Unadjusted Mean Relative Risk (RR) of All-Cause Mortality for Different Categories of Drinkers for Testing Publication Bias and Heterogeneity of RR Estimates From Included Studies

eTable 3. Mean Relative Risk (RR) Estimates of All-Cause Mortality Due to Alcohol Consumption up to 2022 for Subgroups (Cohorts Recruited 50 Years of Age or Younger and Followed up to 60 Years of Age)

This supplemental material has been provided by the authors to give readers additional information about their work.

© 2023 Zhao J et al. *JAMA Network Open.*

eAppendix. Methodology of Meta-analysis on All-Cause Mortality and Alcohol Consumption

Methods

The present study is designed to update the systematic reviews and meta-analyses performed by Stockwell et al. (2016)[1] by including studies published up to July 2021. There was a total of 107 cohort studies after inclusion of 20 new studies meeting inclusion criteria. The study protocol was registered in advance on the Open Science Framework.[2]

Inclusion criteria

Language: This review requires studies to be published in English but did not exclude studies of no–European origin.

Publication type: This review included all original research manuscripts published in peer–reviewed journals. Studies examined the relationship between all–cause mortality and alcohol consumption among human populations in cohort studies (see Stockwell et al, 2016).[1]

Types of study: The review considered all retrospective or prospective cohort studies evaluating the relationship between alcohol consumption and all–cause mortality risk.

Types of participants: This review considered all cohort studies that involve human subjects of any age. The sample must include alcohol users. All genders, age groups and subjects from any racial, ethnic, cultural or religious groups are eligible for inclusion, regardless of location.

Types of outcome measures: The primary outcomes of interest are all-cause mortality.

Types of exposure: Exposure of interest is alcohol consumption. Studies which have at least three categories of alcohol consumption reported were included.

When studies assess usual or typical drinking patterns over a month or a week, it is assumed that individuals classified as abstainers by this method and used as the reference group would

© 2023 Zhao J et al. *JAMA Network Open.*

include occasional drinkers (i.e., drinkers who typically consumed less than weekly or monthly). Abstainers also included former drinkers i.e. those who have not drunk any alcohol in the past year when they were interviewed in each of individual studies but who have had at least one drink of alcohol in their lives. We coded a drinking measure as minimally “adequate” if both quantity and frequency of drinking were assessed for a period of at least one week or lifetime alcohol use was measured.

Exclusion criteria

Publication type: Only original cohort studies were included. All other study designs were excluded as well as narrative reviews, letters, editorials, commentaries, unpublished manuscripts, dissertations, government reports, books and book chapters, conference proceedings, meeting abstracts, lectures and address, and consensus development statements including guideline statements.

Types of participants: Cohort studies that involve human subjects selected on the basis of having an illness and/or a heavy/problematic/dependent pattern of drinking were excluded. Studies that were based on the general population but included those who had coronary heart disease (CHD) or other conditions were not excluded.

Search strategy

The systematic review followed the Preferred Reporting Items for Systematic Reviews and Meta–Analyses (PRISMA) guidelines.[3] Two addiction information scientists from the Canadian Centre on Substance Use and Addiction (CCSA) implemented the first stage of the protocol by repeating the exact search strategies used to identify relevant articles utilised by Stockwell et al. (2016) [1] for articles quick published between January 1, 2015 and July 31, 2021. The electronic databases PUBMED and Web of Sciences were systematically searched from January 1, 2015 in

order to capture the most recent literature and include all relevant published studies. The reference lists of papers meeting the eligibility criteria were screened for additional potentially relevant papers that may have been missed by our electronic searches. We identified all potentially relevant articles regardless of language by searching Pubmed and Web of Science, through reference list cross–checking including those of previous meta–analyses and incorporating publications up to July 31, 2021. We used the following key words and subject headings to identify relevant articles in electronic databases: [mortality OR death] AND [alcohol OR consumption OR ethanol OR alcohol drinking] AND [cohort OR prospective OR longitudinal]. We searched for both all-cause mortality studies and studies of deaths from coronary cohort disease (CHD) and alcohol use but conducted meta-analyses on alcohol use and all-cause mortality.

Study selection

Included studies were: original cohort studies published in English, with mortality from any conditions, at least three levels of alcohol consumption quantified for human subjects of all ages. There were 107 studies that satisfied the criteria for the meta–analysis on all-cause mortality and alcohol use (eFigure 1). Among 107 studies, there were 87 studies that have been used in the study by Stockwell et al (2016)[1] and 20 newly-identified studies (eTable 1).[4-23]

Insert eFigure 1 and eTable 1 about here

Two reviewers from CCSA read the titles of all the citations and abstracts retrieved from the electronic database searches and removed all citations that are clearly not related to the studies of the relationship between all-cause mortality and alcohol consumption. The screening further involved abstract review. Full–text articles were obtained for all abstracts except for those that clearly did not meet eligibility criteria. The investigators were consulted in the event of any

© 2023 Zhao J et al. *JAMA Network Open.*

disagreement. Two investigators (TS and JZ) independently evaluated the studies selected for inclusion.

Data extraction

Three reviewers (JZ, FA and JC) reviewed all eligible papers to extract and code data independently from all studies fulfilling the inclusion criteria, and the disagreements were resolved by discussion with the PI and investigators. The original codebook prepared and used by Stockwell et al.[1] has been refined and updated to provide more detailed classifications of the type of reference groups employed, the methods of quantifying alcohol consumption, and of defining consumption levels and identifying higher quality studies that are less likely to suffer from lifetime selection biases. Each study was coded with reference to the standardized codebook and under the supervision of the lead investigator. The latest report was coded if multiple reports have been issued from the same dataset unless different populations are considered (e.g. males versus females). The coding of all variables in the meta–dataset was double–checked by the investigators (TS and JZ). The types of data to be extracted include (1) outcome, hazards ratio, rate ratio or rate of all-cause mortality by alcohol drinking or not drinking groups; (2) measures of alcohol consumption that can be converted into the amount of ethanol per day; (3) study characteristics, including cohort ages at recruitment and follow-up years, country in which the studies were conducted and published year of the studies; (4) types of misclassification error of alcohol consumers and abstainers; (5) controlled variables in individual studies. Specific variables were defined and specified below. Alcoholic ‘drinks” were converted into grams per day based on country-specific definitions if not otherwise defined.[24,25]

Outcomes: The review examined mortality from all causes due to alcohol consumption. The outcome variable of interest is defined as the presence or absence of all-cause mortality.[26]

© 2023 Zhao J et al. *JAMA Network Open.*

Hazard ratios and rate ratio estimates of mortality in individual studies were used as the relative risk (RR) estimates. Where studies only report mortality rates, these were converted to RR estimates.[27] When "abstainers" specified in original studies are not the reference category, the RR values were recalculated with "abstainers" as the reference group.[1,28]

Measures of alcohol consumption: The primary exposure variable is level of daily alcohol consumption in grams of ethanol assessed at (i) baseline and, if available, (ii) over a lifetime and compared with a reference group of variously defined "non–drinkers" or "abstainers". When studies do not define the grams of alcohol per unit or drink, we used 8 g/unit for the United Kingdom; 10 g/drink for Australia, Austria, France, Greece, Hungary, Ireland, Netherlands, New Zealand, Poland, Spain, Sweden; 11 g/drink for Finland; 12 g/drink for Denmark, Germany, Italy, South Africa and Switzerland; 13.45 g/drink for Canada; 14 g/drink for the United States; 12.5 g/drink for China, 19.75 g/drink for Japan and 12 g/drink for other countries.[24,25] Alcohol use was converted into grams per day using the mid–points of reported categories to estimate mean values. For open–ended top categories (e.g. 6+ drinks/day) we added three–quarters of the range of the next lowest category to the lower bound (e.g. if 3 to 5 drinks this would be 6+(5–3)*0.75=7.5).[29]

Study characteristics: (1) study name, authors and publication date; (2) geographic origin, i.e., in which countries individual studies were conducted; (3) characteristics of study population including age (youngest age, median age and oldest age), sex (male only, females only or both), study and publication years, years of follow–up, health status (no exclusion for illness or ill people separated in analyses, CHD diagnoses excluded from analysis or separated, CHD or other illnesses excluded from analysis), drinking patterns (daily quantity and frequency of drinking over different time periods from past week up to over lifetime).

© 2023 Zhao J et al. *JAMA Network Open.*

Types of misclassification error of abstainer/drinker categories: classification of studies on basis of whether the abstainer category includes former or occasional drinkers: a) abstainers contaminated by both occasional drinkers and former drinkers, b) abstainers contaminated by occasional drinkers only, c) abstainers contaminated by former drinkers only and d) abstainers not contaminated by either occasional drinkers or former drinkers. The study defined that lifetime nondrinkers or lifetime abstainers[30] were those who never drank one drink in their lifetime and former drinkers were those who drank at least one drink per day but now completely stopped.[1,28] Active drinkers or current drinkers were those who drink 1.3 grams per day at baseline of the studies. When the "abstainers" were considered as the reference group in the original studies contaminated by former drinkers or active drinkers, usually occasional or light drinkers, the RR estimates for drinkers were considered the biased estimates and thus an adjustment was needed.

Following Fillmore et al (2006)[28] and Stockwell et al (2016),[1] lifetime abstention was strictly defined as zero consumption, or never drank one drink and did not include studies with any level of occasional lifetime or past year drinking (e.g. less than 12 drinks or "rarely" or "hardly ever" drinking). Our rationale for this strict criterion is that self–reported infrequent drinkers have been shown to greatly under report their personal consumption.[31,32]

The studies that removed former drinkers from the abstainer group were coded as to whether these former drinkers were then reallocated to active drinking categories to avoid reverse former drinker bias i.e. by selecting out sick people from the active drinking categories being assessed. Controlled covariates: studies were coded for which variables are controlled in final models, these include age, gender, indications of heart problems (or excluded from analysis), diagnosed or reported diabetes (or excluded from analysis), general health (or excluded from analysis), mental health, disability or limitations in daily living, smoking, marital status, social

© 2023 Zhao J et al. *JAMA Network Open.*

status, exercise, race, region of country, clinical currently having health insurance, attending religious services, number of live births, calendar year of occurrence/diagnosis, relative weight, former drinking, type of housing, social support, period of time preceding the interview, follow–up duration.

Strategy for data analysis

Descriptive analysis was performed to describe the number and percentage of studies and the RR estimates by subgroups from included studies. Linear regression[27] was used to estimate mean RRs for any drinking by subgroups such as median age or sex of the included studies and identify potential confounders to be included in multivariable mixed linear regression models that were used to estimate adjusted RR due to drinking alcohol.

Publication bias was assessed through visual inspection of the funnel plot of log–RR of all-cause mortality due to alcohol consumption against the inverse standard error of log–RR[27] and the Egger's linear regression method.[33] We also plotted forest graphs of log-RR of all-cause mortality for any level drinking to assess heterogeneity among newly-searched studies.[34] We assessed between–study heterogeneity of RRs from all included studies using Cochran's Q[34] and the I^2 statistic.[35] If heterogeneity was detected, mixed effects models were used to obtain the summarized RR estimates.

Mixed effects regression analyses were performed in which drinking groups and control variables were treated as fixed effects with a random study effect.[36] The dependent variable was the natural log of the relative risk estimated using the rate ratio or hazard ratio of each drinking group in relation to the abstainer category to deal with the skewed distribution of the estimates.[27] All analyses were weighted by the inverse of the estimated variance of the natural log relative risk. Variance was estimated from reported standard errors, confidence intervals or number of deaths.

© 2023 Zhao J et al. *JAMA Network Open.*

The weights for each individual study were created using the inverse variance weight scheme and used in mixed regression analysis in order to get maximum precision for the main results of the meta–analysis.[27]

Drinking level in each study group were examined in terms of pre–defined specific consumption levels. Drinking categories were defined and reclassified if necessary as (1) lifetime occasional drinkers (0.02–0.33 grams per day, only two RR estimates that were excluded from meta-analysis), (2) former drinkers now completely abstaining, (3) current occasional drinkers, up to one drink (=10 grams of ethanol) per week (<1.30 grams per day or (<9.1 grams per week); (4) low volume drinkers, up to 2 drinks or 1.30–24 grams per day; (5) medium volume drinkers, up to 4 drinks or 25–44 grams per day; (6) high volume drinkers, up to 6 drinks or 25–64 grams per day; (7) highest volume drinkers, 6 drinks or 65 grams or more per day. All studies have an open–ended heavier drinking group, i.e., with no upper limit of quantity consumed per day for responses accepted as valid.

The analysis tested interactions of alcohol drinking with median age or sex of the study populations in order to identify possible effect modification. That is whether the risk significantly differed by genders or median ages of study populations. As a result, further analyses were conducted separately for men and women, median age ≤55 and 56+. An additional analysis was performed based on studies of cohorts 50 years old or younger recruited and followed up in health records until a median age of at least 60 years (eTable 3).

The analyses adjusted for the confounding effects of median age and sex of study populations, drinker biases, country where a study was conducted, follow-up years of the study cohorts and year of study publication. Other covariates available were selected for inclusion on empirical grounds based on p–values of bivariable tests of the log–RR and each covariate, and

significant correlations with other variables. Based on bivariable analysis of the dataset, any variable whose bivariable test had a p–value <0.20 was considered as a candidate for the multivariable regression analyses of the log–RR of all-cause mortality and alcohol consumption.[37] We also considered the change-in-estimate, i.e., whether crude and adjusted estimates differ by 10%.[38] We detected the effects and multicollinearity by exploring the correlation matrix.[39,40] If two or more covariates are highly correlated (coefficient >0.80), the one with the lowest p–value from the univariable test were included in the multivariable regression analyses. All selected covariates were included in models to adjust for their potential confounding effects. This approach was employed because it is possible for individual variables to not exhibit strong confounding, but when taken collectively, considerable confounding may be present in the data.[37]

All significance tests assume two–tailed P values or 95% CIs. All statistical analyses were performed using SAS 9.4 and the SAS PROC MIXED procedure was used to model the log–transformed RR of all-cause mortality due to alcohol consumption after adjusting for potential confounding effects of various factors.[41] The mean RR estimates of natural logarithmic transformation were converted to exponential scales.

eReferences

1. Stockwell T, Zhao JH, Panwar S, Roemer A, Naimi T, Chikritzhs T. Do "Moderate" Drinkers Have Reduced Mortality Risk? A Systematic Review and Meta-Analysis of Alcohol Consumption and All-Cause Mortality. J Stud Alcohol Drugs 2016; 77(2): 185-98.

2. Stockwell TR, Zhao J, Churchill S, et al. An updated systematic review and new meta analyses of studies on alcohol use and mortality risk that have reduced risk of lifetime selection biases. Open Science Framework Protocol (https://osf.io/tnhd3/); 2021.

3. Moher D, Liberati A, Tetzlaff J, Altman DG, Grp P. Preferred Reporting Items for Systematic Reviews and Meta-Analyses: The PRISMA Statement. Plos Med 2009; 6(7).

4. Kaprio J, Latvala A, Rose R. Longitudinal Patterns of Alcohol Use and Mortality - a 40 Year Follow-up of the Finnish Twin Cohort. Eur Neuropsychopharm 2019; 29: S804-S.

© 2023 Zhao J et al. *JAMA Network Open.*

5. Dai J, Mukamal KJ, Krasnow RE, Swan GE, Reed T. Higher usual alcohol consumption was associated with a lower 41-y mortality risk from coronary artery disease in men independent of genetic and common environmental factors: the prospective NHLBI Twin Study. American Journal of Clinical Nutrition 2015; 102(1): 31-9.

6. Bobak M, Malyutina S, Horvat P, et al. Alcohol, drinking pattern and all-cause, cardiovascular and alcohol-related mortality in Eastern Europe. European Journal of Epidemiology 2016; 31(1): 21-30.

7. Licaj I, Sandin S, Skeie G, Adami HO, Roswall N, Weiderpass E. Alcohol consumption over time and mortality in the Swedish Women's Lifestyle and Health cohort. Bmj Open 2016; 6(11).

8. Almeida OP, McCaul K, Hankey GJ, Yeap BB, Golledge J, Flicker L. Excessive alcohol consumption increases mortality in later life: a genetic analysis of the health in men cohort study. Addict Biol 2017; 22(2): 570-8.

9. Luksiene D, Tamosiunas A, Virviciute D, Radisauskas R. The Prognostic Value of Combined Smoking and Alcohol Consumption Habits for the Estimation of Cause-Specific Mortality in Middle-Age and Elderly Population: Results from a Long-Term Cohort Study in Lithuania. Biomed Res Int 2017; 2017.

10. Perreault K, Bauman A, Johnson N, Britton A, Rangul V, Stamatakis E. Does physical activity moderate the association between alcohol drinking and all-cause, cancer and cardiovascular diseases mortality? A pooled analysis of eight British population cohorts. Brit J Sport Med 2017; 51(8): 651-+.

11. Syden L, Landberg J. The contribution of alcohol use and other lifestyle factors to socioeconomic differences in all-cause mortality in a Swedish cohort. Drug and Alcohol Review 2017; 36(5): 691-700.

12. Kunzmann AT, Coleman HG, Huang WY, Berndt SI. The association of lifetime alcohol use with mortality and cancer risk in older adults: A cohort study. Plos Med 2018; 15(6).

13. Ortola R, Garcia-Esquinas E, Lopez-Garcia E, Leon-Munoz LM, Banegas JR, Rodriguez-Artalejo F. Alcohol consumption and all-cause mortality in older adults in Spain: an analysis accounting for the main methodological issues. Addiction 2019; 114(1): 59-68.

14. Saito E, Inoue M, Sawada N, et al. Impact of Alcohol Intake and Drinking Patterns on Mortality From All Causes and Major Causes of Death in a Japanese Population. J Epidemiol 2018; 28(3): 140-8.

15. Keyes KM, Calvo E, Ornstein KA, et al. Alcohol Consumption in Later Life and Mortality in the United States: Results from 9 Waves of the Health and Retirement Study. Alcohol Clin Exp Res 2019; 43(8): 1734-46.

16. Rosella LC, Kornas K, Huang A, Grant L, Bornbaum C, Henry D. Population risk and burden of health behavioral-related all-cause, premature, and amenable deaths in Ontario,

© 2023 Zhao J et al. *JAMA Network Open.*

Canada: Canadian Community Health Survey-linked mortality files. Ann Epidemiol 2019; 32: 49-57.

17. Daya NR, Rebholz CM, Appel LJ, Selvin E, Lazo M. Alcohol Consumption and Risk of Hospitalizations and Mortality in the Atherosclerosis Risk in Communities Study. Alcohol Clin Exp Res 2020; 44(8): 1646-57.

18. Jankhotkaew J, Bundhamcharoen K, Suphanchaimat R, et al. Associations between alcohol consumption trajectory and deaths due to cancer, cardiovascular diseases and all-cause mortality: a 30-year follow-up cohort study in Thailand. Bmj Open 2020; 10(12).

19. van de Luitgaarden IAT, Schrieks IC, Kieneker LM, et al. Urinary Ethyl Glucuronide as Measure of Alcohol Consumption and Risk of Cardiovascular Disease: A Population-Based Cohort Study. J Am Heart Assoc 2020; 9(7).

20. Rojas NBA, Lacey B, Simadibrata DM, et al. Alcohol consumption and cause-specific mortality in Cuba: prospective study of 120 623 adults. Eclinicalmedicine 2021; 33.

21. Di Castelnuovo A, Costanzo S, Bonaccio M, et al. Alcohol intake and total mortality in 142 960 individuals from the MORGAM Project: a population-based study. Addiction 2022; 117(2): 312-25.

22. Martinez-Gonzalez MA, Barberia-Latasa M, de Rojas JP, Rodriguez LJD, Sanchez AG. Alcohol and early mortality (before 65 years) in the 'Seguimiento Universidad de Navarra' (SUN) cohort: does any level reduce mortality? Brit J Nutr 2022; 127(9): 1415-25.

23. Zhang XY, Liu Y, Li SS, et al. Alcohol consumption and risk of cardiovascular disease, cancer and mortality: a prospective cohort study. Nutr J 2021; 20(1).

24. Tumer C. How much alcohol is in a 'standard drink'? An analysis of 125 studies. British Journal of Addiction 1990; 85(9): 6.

25. International Alliance for Responsible Drinking. Drinking Guidelines: General Population. https://iard.org/science-resources/detail/drinking-guidelines-general-population/Accessible September 10, 2022. International Alliance for Responsible Drinking; 2019.

26. WHO. International statistical classification of diseases and related health problems - 10th Revision. Geneva: WHO. Available from URL: http://www.who.int/classifications/icd/ICD10Volume2_en_2010.pdf. Accessible 10 September 2022; 2010.

27. Woodward M. Epidemiology Study design and data analysis. New York: Chapman & Hall/CRC; 2000.

28. Fillmore KM, Kerr WC, Stockwell T, Chikritzhs T, Bostrom A. Moderate alcohol use and reduced mortality risk: Systematic error in prospective studies. Addiction Research & Theory 2006; 14(2): 101-32.

© 2023 Zhao J et al. *JAMA Network Open.*

29. Roerecke M, Rehm J. The cardioprotective association of average alcohol consumption and ischaemic heart disease: a systematic review and meta-analysis. Addiction 2012; 107(7): 1246-60.

30. WHO. The Global Health Observatory - Explore a world of health data. In: WHO, editor. Global Health Observatory data repository. Geneva, Switzerland: WHO. Accessible on October 28, 2022: https://www.who.int/data/gho/indicator-metadata-registry/imr-details/460; 2022.

31. Stockwell T, Zhao JH, Macdonald S. Who under-reports their alcohol consumption in telephone surveys and by how much? An application of the 'yesterday method' in a national Canadian substance use survey. Addiction 2014; 109(10): 1657-66.

32. Ye Y, Bond J, Cherpitel CJ, Stockwell T, Macdonald S, Rehm J. Risk of Injury Due to Alcohol Evaluating Potential Bias Using the Case-Crossover Usual-Frequency Method. Epidemiology 2013; 24(2): 240-3.

33. Egger M, Smith GD, Schneider M, Minder C. Bias in meta-analysis detected by a simple, graphical test. British Medical Journal 1997; 315(7109): 629-34.

34. Cochran WG. The Combination of Estimates from Different Experiments. Biometrics 1954; 10(1): 101-29.

35. Higgins JPT, Thompson SG. Quantifying heterogeneity in a meta-analysis. Statistics in Medicine 2002; 21(11): 1539-58.

36. Normand SLT. Meta-analysis: Formulating, evaluating, combining, and reporting. Statistics in Medicine 1999; 18(3): 321-59.

37. Hosmer DW, Lemeshow S. Applied logistic regression. New York: Wiley; 2000.

38. Maldonado G, Greenland S. Simulation Study of Confounder-Selection Strategies. American Journal of Epidemiology 1993; 138(11): 923-36.

39. Allison P. When can you safely ignore multicollinearity? . 2012. https://statisticalhorizons.com/multicollinearity (accessed September 10, 2022).

40. Schreiber-Gregory DN. Multicollinearity: wha is it, why should we care, and how can it be controlled? The SAS Global Forum 2017 Conference; 2017; Orlando, Florida: SAS Institute Inc. ; 2017.

41. SAS Institute. SAS/STAT 9.4 user's guide. Cary, NC: SAS Institute Inc. ; 2016.

© 2023 Zhao J et al. *JAMA Network Open.*

eFigure 1. Flowchart of Systematic Search Process for Studies of Alcohol Consumption and Risk of All-Cause Mortality

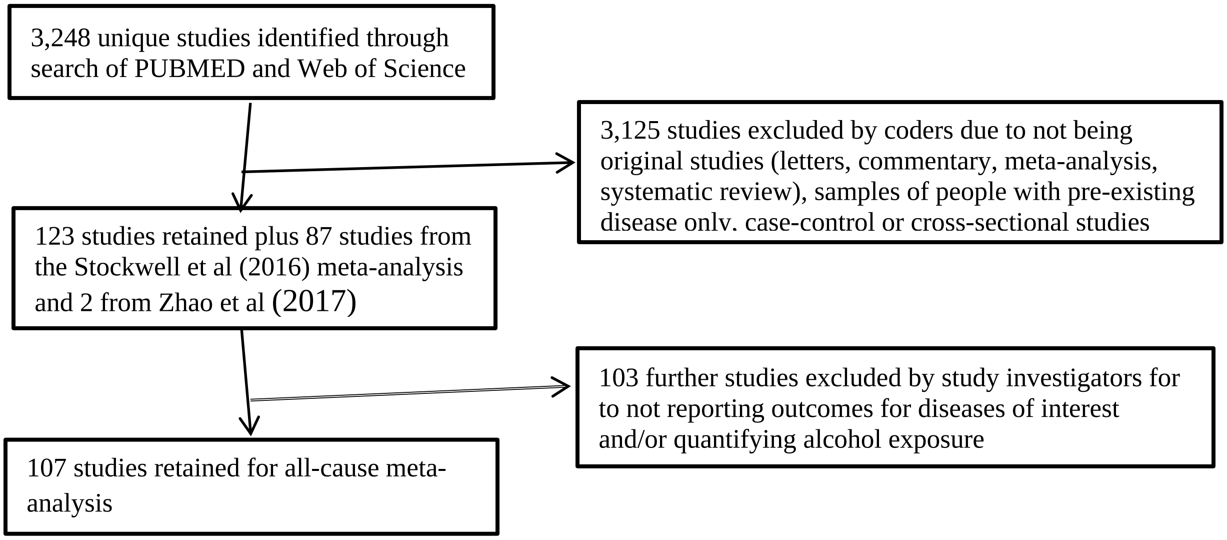

© 2023 Zhao J et al. *JAMA Network Open.*

**eTable 1.** Newly Included 20 Studies (194 Risk Estimates) of All-Cause Mortality and Consumption in 2015 to 2022								
Year	Author	Sex	Country	No. of deaths	No. of subjects	Age range	Follow-up year	Covariates assessed ‡‡
2015	Dai et al	M	US	614	843	42-55	41	1, 5, 9, 10
2016	Bobak et al	M,F	Sweden	2,895	34,259	45-72	6.9	1, 2, 4, 5, 6
2016	Licaj et al	F	Sweden	2,100	33,404	30-49	12	1, 5, 7, 8, 9
2017	Almeida et al	M	Australia	1,329	3,496	70-89	8	1, 7, 9
2017	Luksiene et al	M,F	Lithuania	2,158	6,729	35-64	31	1, 5, 6, 7
2017	Perreault et al	T	UK	5,735	36,370	40-102	9.7	1, 5, 7, 8, 9
2017	Syden et al	T	UK	300	21,064	25-74	5	1, 3, 5, 7, 8, 9, 10
2018	Kunzmann et al	M,F,T	US	9,599	99,654	55-74	8.3	1, 5, 7, 10
2018	Saito et al	M,F	Japan	15,203	102,849	40-69	18.2	1, 3, 5, 7, 8, 10
2019	Kaprio et al †	M,F,T	Finland	11,584	26,070	18-96	36.2	1, 5, 7, 10
2019	Keyes et al	M,F	UK	2,399	7,904	56-99	16	1, 3, 4, 5, 7, 8
2019	Ortola et al	T	Spain	327	3,045	60-99	7.8	1, 3, 4, 5, 7, 8, 9
2020	Daya et al	M,F,T	US	629	12,327	47-73	22.3	1, 3, 4, 5, 6, 7, 8, 9
2020	Jankhotkaew et al	T	Thailand	276	1,961	35-54	30	1, 2, 3, 7, 8, 9
2020	vande Luitgaarden et al	T	Netherlands	724	5,676	28-75	14.1	1, 3, 5, 6, 7, 8
2021	Rojas et al	M,F,T	Cuba	4,355	120,623	35-79	17	1, 4, 5, 6, 7, 9
2021	Di Castelnuovo et al ‡	T	MORGAM	16,907	142,960	18-81	11.8	1, 3, 5, 6, 7, 9
2021	Martinez-Gonzalez et al	M,F	Italy	226	20,272	20-65	12.3	1, 5, 6, 7, 8, 9, 10
2021	Zhang et al	T	China	6,446	83,732	18-90	10	1, 5, 7, 8, 9, 10
Note: † The authors updated the RR estimates in March, 2022. ‡ The MORGAM Project included 10 European countries (Finland, France, Germany, Italy, Lithuania, Norway, Poland, Russia, Sweden, and UK) and Australia. ‡‡ 1: age; 2: sex; 3: social status; 4: race; 5: smoking status; 6: Illness exclusion; 7: body mass index; 8: exercise; 9: heart disease; 10: diet.								

© 2023 Zhao J et al. *JAMA Network Open.*

eFigure 2. Funnel Plot of Log-Relative Risk (In(RR)) of All-Cause Mortality Due to Alcohol Consumption Against Inverse of Standard Error of In(RR)

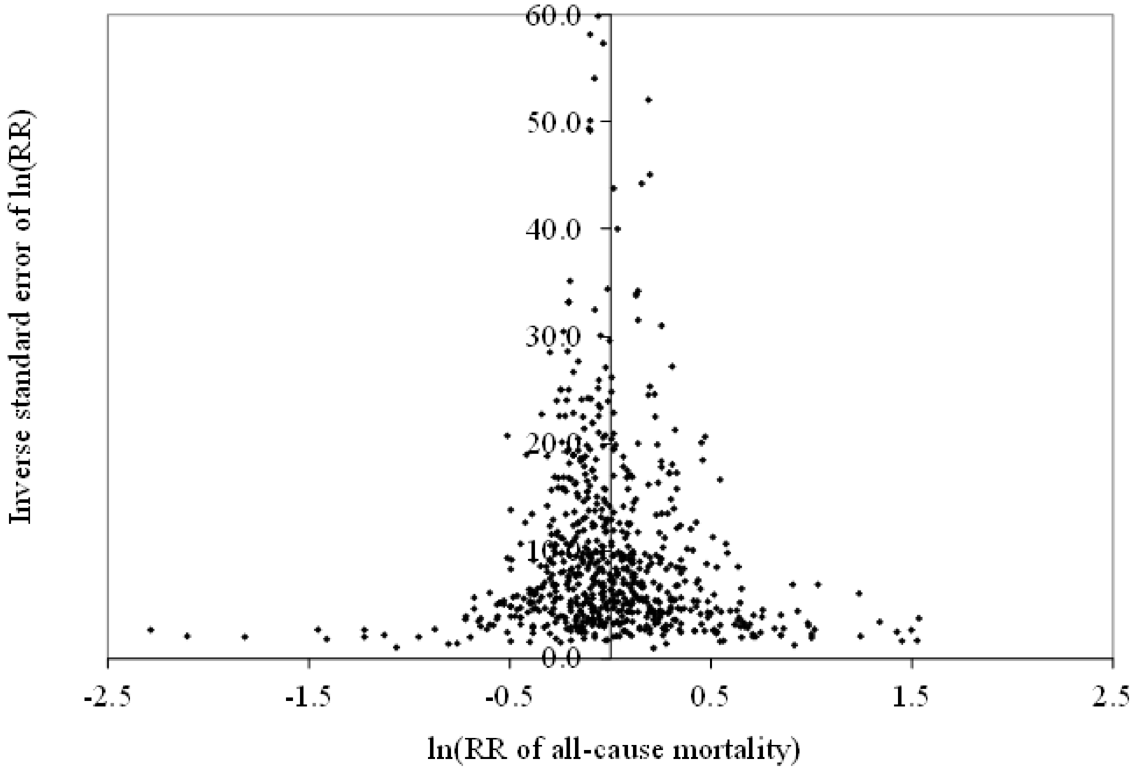

© 2023 Zhao J et al. *JAMA Network Open.*

eFigure 3. Relative Risk (95% CI) of All-Cause Mortality Due to Any Alcohol Consumption Without Any Adjustment for Characteristics of New Studies Published between 2015 and 2022

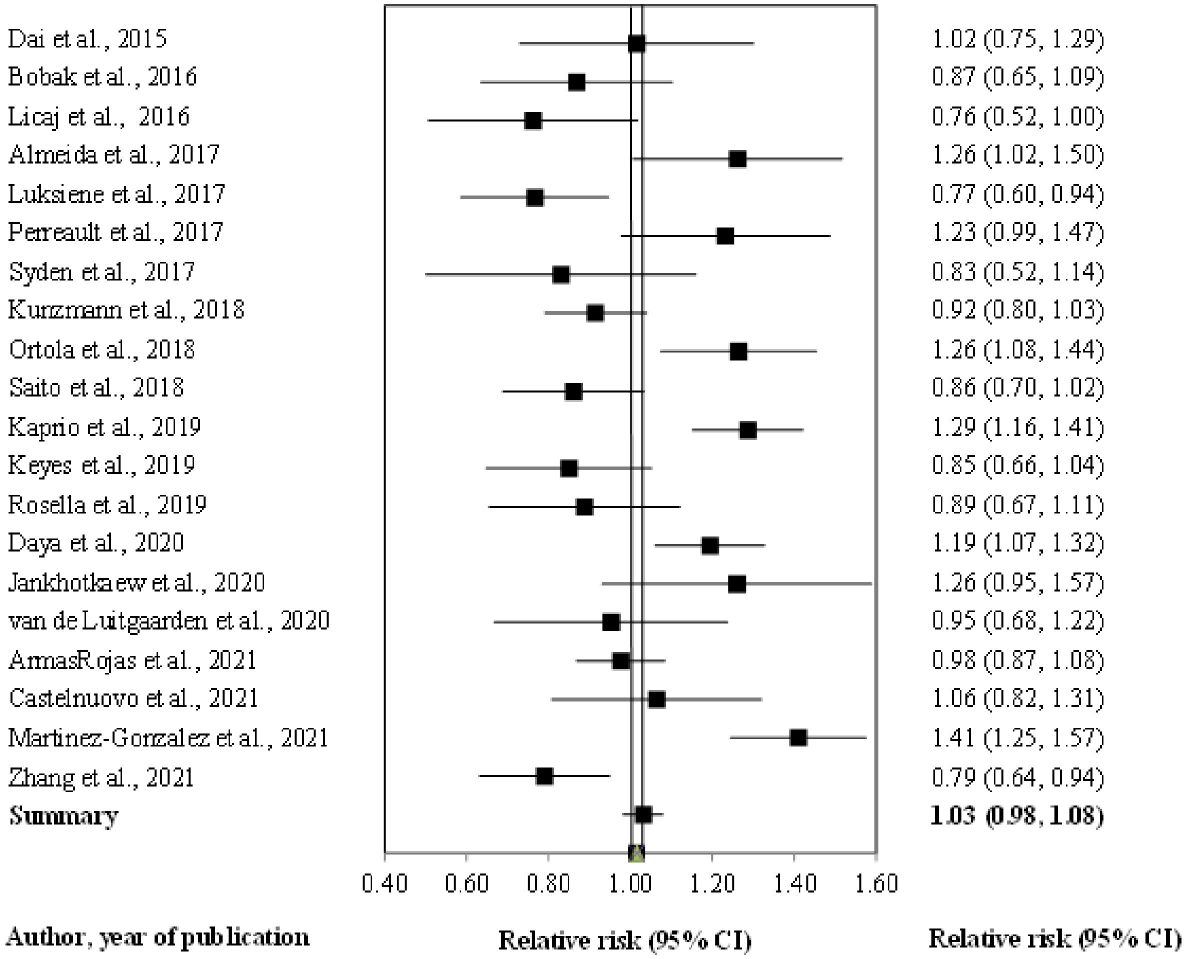

© 2023 Zhao J et al. *JAMA Network Open.*

eFigure 4. Unadjusted, Partially Adjusted, and Fully Adjusted relative Risk (RR) of All-Cause Mortality for Drinkers (vs Nondrinkers), 1980 to 2022

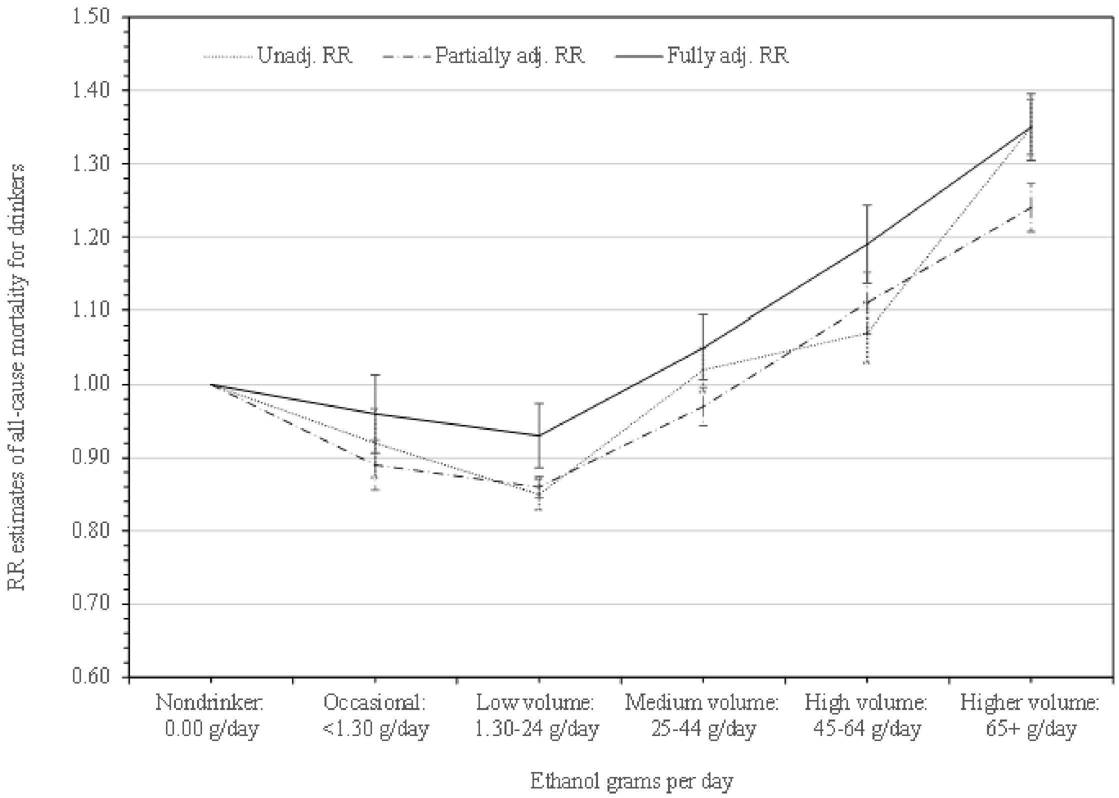

© 2023 Zhao J et al. *JAMA Network Open.*

eTable 2. Statistical Analysis of Unadjusted Mean Relative Risk (RR) of All-Cause Mortality for Different Categories of Drinkers for Testing Publication Bias and Heterogeneity of RR Estimates From Included Studies

Drinking categories	N/n †	Egger's linear regression for publication bias		Tests for heterogeneity	
		Coefficient	ttest P	P (Q statistic)	I^2 (%, 95% CI)
Former drinker	28/56	+0.13	=0.7476	<0.001	60.97 (47.82 – 70.81)
Occasional (<1.30 g/day)	24/57	–0.31	=0.3998	<0.001	68.99 (59.33 – 76.35)
Low (1.30–<25 g /day)	99/306	–0.07	=0.6300	<0.001	73.46 (70.32 – 76.27)
Medium (25–<45 g/day)	80/146	+0.34	=0.1990	<0.001	80.41 (77.27 – 83.12)
High (45–<65 g/day)	52/76	-0.32	=0.4062	<0.001	85.99 (83.09 – 88.39)
Higher (65+ g/day)	45/83	+0.71	=0.0613	<0.001	79.27 (74.66 – 83.03)
Any drinking	107/724	+0.15	=0.2718	<0.001	85.87 (84.99 – 86.71)

Note: † N= Number of studies and n= Number of risk estimates.

© 2023 Zhao J et al. *JAMA Network Open.*

eTable 3. Mean Relative Risk (RR) Estimates of All-Cause Mortality Due to Alcohol Consumption up to 2022 for Subgroups (Cohorts Recruited 50 Years of Age or Younger and Followed Up to 60 Years of Age)

Drinking categories **!!**	N/n †	Unadjusted ‡			Partially-adjusted ‡‡			Fully-adjusted ‡‡‡		
		RR	95% CI	ttest P	RR	95% CI	ttest P	RR	95% CI	ttest P
Abstainer		1.00			1.00			1.00		
Any drinker vs abstainer	26/153	1.10	0.89 – 1.36	0.3078	1.11	0.93 – 1.31	0.1869	**1.35**	**1.14 – 1.59**	**0.0057**
Former drinker vs abstainer	5/9	**1.49**	**1.19 – 1.86**	**0.0005**	**1.18**	**1.04 – 1.33**	**0.0121**	**1.46**	**1.27 – 1.68**	**0.0001**
Active drinker vs abstainer	26/144	1.01	0.95 – 1.07	0.7631	0.96	0.89 – 1.04	0.2980	1.16	1.00 – 1.35	0.0514
Occasional (<1.30 g/day)	4/6	0.93	0.71 – 1.24	0.6060	0.97	0.87 – 1.09	0.6528	**1.21**	**1.06 – 1.37**	**0.0049**
Low volume (1.30–<25 g/day)	20/71	**0.85**	**0.78 – 0.92**	**0.0001**	**0.85**	**0.77 – 0.93**	**0.0005**	1.05	0.94 – 1.17	0.3843
Medium volume (25–<45 g/day)	20/34	**1.15**	**1.03 – 1.29**	**0.0143**	1.02	0.93 – 1.13	0.6670	**1.26**	**1.12 – 1.42**	**0.0002**
High volume (45–<65 g/day)	6/10	0.97	0.78 – 1.19	0.7379	**1.24**	**1.11 – 1.37**	**0.0001**	**1.52**	**1.33 – 1.73**	**0.0001**
Higher volume (65+ g/day)	14/23	**1.34**	**1.16 – 1.53**	**0.0001**	**1.40**	**1.24 – 1.57**	**0.0001**	**1.68**	**1.47 – 1.92**	**0.0001**

Note: !! Study selection is restricted to still younger cohorts (recruited 50 or below 50 years of age, followed up to 60 years of age). † N= Number of studies and n= Number of risk estimates. ‡ Natural log of the relative risk (RR) estimated using the rate ratio or hazard ratio without weighting and adjusting for between-study variation or covariates. ‡‡ Weighted estimates adjusted for between–study variation. ‡‡‡ Weighted estimates adjusted for between-study variation, abstainer biases, sex, country in which a study was conducted, study publication year, drinking pattern and whether studies controlled for heart problem, social status, race, diet, exercise, BMI, and smoking status.

© 2023 Zhao J et al. *JAMA Network Open.*
