# Supplementary material for: Association Between Daily Alcohol Intake and Risk of All-Cause Mortality: A Systematic Review and Meta-analyses
Source: JAMA Netw Open. 2023 Mar 31;6(3):e236185. doi: 10.1001/jamanetworkopen.2023.6185 (PMC10066463; doi:10.1001/jamanetworkopen.2023.6185)
Supplement: Supplement 2. — Data Sharing Statement [file jamanetwopen-e236185-s002.pdf]

Data Sharing Statement

Zhao. Association Between Daily Alcohol Intake and Risk of All-Cause Mortality. *JAMA Netw Open*. Published March 31, 2023. doi:10.1001/jamanetworkopen.2023.6185

Data
Data available: No
